# Cytokines in relation to hCG are significantly altered in asymptomatic women with miscarriage – a pilot study

**DOI:** 10.1186/s12958-018-0411-5

**Published:** 2018-09-28

**Authors:** Alexander Freis, Janina Schlegel, Volker Daniel, Julia Jauckus, Thomas Strowitzki, Ariane Germeyer

**Affiliations:** 1 Department of Gynaecological Endocrinology and Fertility Disorders, University Hospital Heidelberg, INF 440, 69120 Heidelberg, Germany; 2 Transplantation-Immunology, Institute of Immunology, University Hospital Heidelberg, Im Neuenheimer Feld 305, 69120 Heidelberg, Germany

**Keywords:** Miscarriage, Biomarker, Early pregnancy, hCG, Chemokines

## Abstract

**Background:**

Spontaneous abortion is one of the most common complications in early pregnancy. A preventive test to identify women who will experience a miscarriage, even before first symptoms occur, is not established. Activation of maternal immunological tolerance seems to be essential for early fetal development and various cytokines have been described in different stages of pregnancy. Therefore, we aimed to investigate if chemokine levels at the time of pregnancy testing relative to human Choriogonadotropin (hCG) are altered in patients who will experience a miscarriage in this pregnancy.

**Methods:**

We obtained blood samples from 39 women. Dependent on the follow-up, patients with a positive pregnancy test were subsequently divided in two groups: ongoing pregnancy (*n* = 22) and miscarriage (*n* = 17) in this pregnancy. Immunological and endocrine profiling of maternal plasma at the time of pregnancy testing (5th week of gestation) was performed for each group at the time of pregnancy test using Multiplex and ELISA analysis.

**Results:**

hCG was significantly decreased in patients with abortion whereas levels of IL-1ra, MIP-1a and TNF-alpha were significantly increased. GCSF/ IL-1ra-ratio was 1.66-fold increased in patients with ongoing pregnancy. TGF-beta /MIP1a-ratio was significantly 3.45-times higher in patients with miscarriage. Comparing patients with ongoing pregnancy to patients experiencing a miscarriage, we could demonstrate significant alterations of the ratios MIP1a/hCG, IL-1ra/hCG, TNFalpha/hCG, MCP1/hCG, IL-6/hCG, TPO/hCG and TGF-beta1/hCG. The strongest effects were seen for the ratio MIP1a/hCG, IL-1ra/hCG and TNFalpha/hCG.

**Conclusions:**

We have shown that cytokines in relation to hCG after 4 weeks of gestation are significantly altered in women with miscarriage, promising potential as a prognostic biomarker.

## Background

Spontaneous abortion is one of the most common complications in early pregnancy and affects 10–20% of all pregnancies [1–5]. Symptoms include vaginal bleeding, and/or uterine cramping, but only 28% of symptomatic patients are experiencing spontaneous abortions later in pregnancy [6, 7]. In clinical practice, ultrasound is performed to verify the embryos viability [1]. However, as ultrasound cannot determine pregnancy progress, different hormone assessments have been published in order to help predict pregnancy outcome, such as human chorionic gonadotropin (hCG), progesterone, kisspeptin, activin A, activin B, follistatin, CA-125, pregnancy associated plasma protein A (PAPP-A) or macrophage inhibitory cytokine-1 [1, 2, 4, 6, 8–13]. However, their clinical significance remains fair.

Miscarriage occurs in more than 80% in the first 12 weeks of gestation and presents an enormous distress for the patient and challenges the medical professionals, especially as pregnancy outcome is difficult to estimate when the patient presents with the first symptoms [1, 2, 4, 13]. Moreover, patients with a previous abortion present with a higher risk of severe complications in the following pregnancy, such as preeclampsia or preterm birth [2, 14, 15]**.**

The causes of early pregnancy loss are various, including cytogenetic abnormalities, maternal comorbidities (eg. diabetes mellitus, lupus erythematosus), uterine malformations, smoking as well as inadequate placental development [2, 3]**.**

Activation of maternal immunological tolerance seems to be essential for early fetal development and implantation [16] and various cytokines have been described in different stages of pregnancy [17]**.**

For example, lower levels of interleukin 1-receptor antagonist (IL-1ra) prior to embryo transfer were associated with lower pregnancy rates [18] . Higher levels of Thyreoperoxidase (TPO) and lower concentrations of Granulocyte-Colony Stimulating Factor (GCSF) were significantly associated with the risk of spontaneous abortion in women prior to report of miscarriage. Concerning TPO, this effect is not evident starting before approximately 8 weeks of gestation. However, GCSF alone was only predictive when focusing on women with an upcoming miscarriage within 14 days [17]. Furthermore, chemokine ligand 3 (CCL3)/macrophage inflammatory protein 1-alpha (MIP-1a) is recognized as a local chemoattractant for natural killer cells, whose increased levels in decidual tissue are associated with recurrent pregnancy losses in decidual tissue within the first 10 weeks of gestation [19]. In addition, transforming growth factor (TGF) beta-1 is described as an important factor in immunological reactions and immunological tolerance and shows a lower expression in peripheral blood mononuclear cell cultures of women who experience an abortion or biochemical pregnancy, measured at the time before oocyte retrieval. [20]**.** Furthermore, hCG itself, essential in early pregnancy, cause an increase in regulatory T-cells, has effects on cytokine production, such as an increase in IL-1beta- levels [21], and seems to possess an important role as modulator of immune tolerance during pregnancy [22].

A shift between Th1- and Th2- guided immunological response also seems to play an important role in early pregnancy. The cytokine balance towards Th2-cytokines like Interleukin (IL)-6, as well as tumor necrosis factor (TNF)-alpha is considered important in early pregnancy in maternal blood [23, 24] . Various factors have their influence on this balance, eg. CCL2 or MIP-1 is considered to cause a shift towards the Th2-response of decidual leukocytes [25]**.** However, in contrary to this observation, serum levels of CCL-2/macrophage inflammatory protein (MIP-1) were elevated in women with recurrent spontaneous abortion (RSA) after spontaneous abortion occurred [26]**,** whereas other studies did not see any significant alteration [5]**.**

Most of the currently available studies evaluate symptomatic women, therefore we are the first ones trying to develop biomarkers that are usable even before patients become symptomatic in order to identify women who will experience a miscarriage.

The development of a predictive test for miscarriages can be a helpful instrument to reassure patients on one hand, but also to identify patients who will develop a miscarriage on the other hand and therefore not to prolong unnecessary suffering of the patient. Last, but not least, it may someday lead to potential therapies for such patients [11].

## Methods

The prospective pilot study was approved by Heidelberg University Ethical Committee (protocol S-243/2015) and the experimental testing complied with the principles specified in the Declaration of Helsinki.

Blood samples were obtained at the time of pregnancy testing (5th week of gestation) after informed consent from women who underwent ovarian hyperstimulation to perform in vitro fertilization by IVF or ICSI during the period 05/15–05/16 at the University Hospital in Heidelberg, Germany.

Exclusion criteria were autoimmune diseases, essential hypertonia, diabetes mellitus or the intake of confounding medication (e.g. acetylsalicylic acid).

Dependent on the follow-up, patients with a positive pregnancy test were subsequently divided in two groups: (1) patients with ongoing pregnancy (*n* = 22) and (2) patients with miscarriage (missed abortion, abortus incompletus or abortus completus, *n* = 17) in the first trimester of this pregnancy.

Immunological and endocrine profiling of maternal plasma of women with and without miscarriage was performed for each group at the time of pregnancy testing, in order to predict pregnancy outcome.

### Determination of cytokine levels

IL-6, TNFalpha, (Luminex Performance Assay, Human High Sensitivity Cytokine Base Kit A; R&D systems, Wiesbaden, Germany), IL-1ra, CCL2/MCP-1, CCL3/MIP-1 alpha, G-CSF, Thrombopoietin/Tpo (Human Luminex Performance Assay Base Kit, Panel A; R&D systems, Wiesbaden, Germany), TGF-beta 1 (Luminex Performance Assay 3-plex Kit; R&D systems, Wiesbaden, Germany) were determined in 39 samples using Multiplex and ELISA analysis. Assays were performed according to the instructions of the manufacturer. Measurements were performed as pg/ml and values given as mean+/− SEM.

### Determination of hCG-levels

hCG-levels (mIU/ml, mean+/− SEM) were measured routinely in clinical practice at the time of pregnancy testing from central laboratory, University Hospital Heidelberg, Germany.

### Statistics

Statistical analysis was performed with SPSS® Version 24, IBM, Armonk, USA. *p* ≤ 0.05 was considered to be significant.

## Results

There was no significant difference in age (33.64 ± 6.49 years vs. 33.94 ± 4.4 years), nor in body mass index in group 1 and 2 with 23.81 ± 4.29 kg/m^2^ in group 1 vs. 26.48 ± 5.27 kg/m^2^ in group 2 (Table 1) or transfer day (Table 2).

**Table 1:** Patient characteristics. There was no significant difference in age or BMI between the two groups. All values are given in mean ± STD

	Ongoing pregnancy	Abortion	*p*-value
Age (years)	33.64 ± 6.49	33.94 ± 4.4	0.87
BMI (kg/m2)	23.81 ± 4.29	26.48 ± 5.27	0.12

**Table 2:** Embryo quality and day of embryo transfer

Nr	Abortion	Ongoing pregnacy	Embryo quality	Embryo transfer
1	no	yes	6C, 8B	day 3
2	no	yes	4AA, 4AA	day 5
3	no	yes	4AB, 3AA	day 5
4	no	yes	2A, 4B	day 2
5	no	yes	Morula, blastocyst 1	day 5
6	no	yes	4BA, blastocyst 1	day 5
7	no	yes	4AA hatch	day 5
8	no	yes	5B	day 2
9	no	yes	Blastocyst 1	day 5
10	no	yes	9B	day 3
11	no	yes	4AB, 3AB	day 5
12	no	yes	4AA, 4AA	day 5
13	no	yes	12B	day 4
14	no	yes	3BA	day 5
15	no	yes	Morula	day 4
16	no	yes	4AB, 3AB	day 5
17	no	yes	Blastocyst 1	day 5
18	no	yes	Blastocyst 1	day 4
19	no	yes	Morula, Morula	day 4
20	no	yes	4A	day 2
21	no	yes	6C, 4B	day 3
22	no	yes	4BB, 4BB	day 5
23	yes	no	Blastocyst 1, blastocyst 2	day 4
24	yes	no	3AB, blastocyst 1	day 5
25	yes	no	3BB	day 5
26	yes	no	4AA, 4AA	day 5
27	yes	no	4B	day 2
28	yes	no	11A	day 4
29	yes	no	Blastocyst 1, blastocyst 2	day 5
30	yes	no	4A, 4A	day 2
31	yes	no	4AA, 3AA	day 5
32	yes	no	2A	day 2
33	yes	no	4AA, Blastocyst 1	day 5
34	yes	no	8A, 8C	day 5
35	yes	no	8B, 7C	day 2
36	yes	no	Blastocyst 1, blastocyst 2	day 5
37	yes	no	4BA, 3AA	day 4
38	yes	no	4AA, 4AA	day 3
39	yes	no	9C, 5B, 8C	day 2

hCG-results and immunological profiles of patients with ongoing pregnancy (*n* = 22, Nr. 1–22) and miscarriage (*n* = 17, Nr. 23–39) were analysed. Quantitative difference in GCSF-, MCP-1-, TPO- or IL-6-expression showed a trend towards a change in values, however did not reach statistical significance.

hCG levels, were significantly decreased in patients with miscarriage compared to those with ongoing pregnancy (151.75 ± 25.29 IU/l vs. 351.27 ± 111.02 IU/l, *p* < 0.05) However, they were within normal limits and therefore not predictive for pregnancy outcome.

Similarly, levels of IL-1ra (655.80 ± 78.80 pg/ml vs. 398.69 ± 32.73 pg/ml, *p* < 0.01), MIP-1a (73.74 ± 9.91 pg/ml vs. 34.20 ± 8.25 pg/ml, *p* < 0.01) and TNF-alpha (5.11 ± 0.40 pg/ml vs. 4.00 ± 0.26 pg/ml, *p* < 0.05) were significantly increased.

In order to get a potentially more stable predictor, we investigated if the relative expressions of immunological factors among themselves show a significant alteration.

Here, we found that the ratio of GCSF to IL-1ra was 1.66-fold decreased (*p* < 0.05) in patients suffering from miscarriage (0.043 ± 0.005) compared to patients with ongoing pregnancy (0.072 ± 0.009; *p* < 0.05). In addition, the ratio of MIP1a to TGF-beta was 3,45-times higher in patients with miscarriages (0.012 ± 0.005) compared to patients without ongoing pregnancy (0.004 ± 0.001; *p* < 0.05) (data are shown in Table 3). The other relative expressions of the different cytokines analysed did not show significant changes.

**Table 3:** Significant alterations of relative immunological profile. GCSF, IL-1ra, MIP1a and TGF-beta were assessed in pg/ml

Ratio	GCSF/IL-1ra	MIP1a /TGF-beta 1
mean ± SEM (ongoing pregnancy)	0.072 ± 0.009	0.004 ± 0.001
mean ± SEM (abortion)	0.043 ± 0.005	0.012 ± 0.005
Ratio	1.657	0.293
p-value	< 0.05	< 0.05

Due to the observation, that patients with miscarriage showed lower levels of hCG and increased levels in immunological factors at the same time, we investigated if we get a higher predictive value, if we analyse the relative expression of immunological factors compared to hCG (Table 4).

**Table 4:** Alterations in immunological profile relative to hCG-levels. MIP1a, IL-1ra, TNF alpha, MCP1, IL-6, TPO and TGF-beta1 were assessed in pg/ml, hCG in mIU/ml

Ratio	MIP1a (pg/ml)/ hCG (mIE/ml)	IL-1ra (pg/ml)/ hCG (mIE/ml)	TNF alpha(pg/ml)/ hCG (mIE/ml)	MCP1(pg/ml)/ hCG (mIE/ml)	IL-6 (pg/ml)/ hCG (mIE/ml)	TPO (pg/ml)/ hCG(mIE/ml)	TGF-beta1 (pg/ml)/ hCG(mIE/ml)
mean ± SEM (ongoing pregnancy)	0.16 ± 0.04	2.22 ± 0.72	0.02 ± 0.01	0.5 ± 0.16	0.007 ± 0.002	2.50 ± 0.70	68.04 ± 22.37
mean ± SEM (abortion)	1.02 ± 0.38	7.83 ± 2.30	0.07 ± 0.02	1.44 ± 0.40	0.016 ± 0.003	5.47 ± 1.31	149.35 ± 33.89
Ratio	0.15	0.28	0.29	0.35	0.44	0.45	0.45
p-value	< 0.05	< 0.05	< 0.05	< 0.05	< 0.05	< 0.05	< 0.05

Comparing patients with ongoing pregnancy to patients experiencing a miscarriage, we could demonstrate significant alterations of the ratios MIP1a/hCG (0.16 ± 0.04 pg/mIU vs. 1.02 ± 0.38 pg/mIU, *p* < 0.05), IL-1ra/hCG (2.22 ± 0.72 pg/mIU vs. 7.83 ± 2.30 pg/mIU, *p* < 0.05), TNFalpha /hCG (0.02 ± 0.01 pg/mIU vs. 0.07 ± 0.02 pg/mIU, *p* < 0.05), MCP1/hCG (0.50 ± 0.16 pg/mIU vs. 1.44 ± 0.40 pg/mIU, *p* < 0.05), IL-6/hCG (0.007 ± 0.002 pg/mIU vs 0.016 ± 0.003 pg/mIU, *p* < 0.05), TPO/hCG (2.50 ± 0.70 pg/mIU vs. 5.47 ± 1.31 pg/mIU, *p* < 0.05) and TGF-beta1/hCG (68.04 ± 22.37 pg/mIU vs. 149.35 ± 33.89 pg/mIU, *p* < 0.05).

Altogether, we observe a significant alteration towards the immunological factor in relation to hCG levels in patients, who experience a miscarriage in their course of pregnancy.

The strongest effects were seen for the ratio MIP1a/hCG (ratio 0.15) for patients with ongoing pregnancy compared to patients with abortion, IL-1ra (ratio 0.28) and TNFalpha (ratio 0.29).

## Discussion

Spontaneous abortion is one of the most common complications in early pregnancy and affects 10–20% of all pregnancies [1–5]**.**

There is currently no successful therapeutical approach established, although various approaches have been tested [5, 27]**.**

The development of an early-screening test to identify patients who are at risk to suffer from abortion in the actual pregnancy can be useful in manyfold ways: a miscarriage presents an enormous distress for the patient and a predictive test with a negative result could be used to reassure anxious patients [1, 2, 4, 5, 13]**.** On the other hand, a predictive test with a positive result can warn the patients in a very early stage of pregnancy [5], and will prohibit unnecessary prolongation of the current pregnancy by supplementation of high doses of progesterone, as progesterone prevents bleeding.

Most of the studies trying to establish biomarkers to follow this approach include symptomatic women at a later stage of pregnancy. One of the most interesting aspects concerning our data is the fact that we discovered in total 3 absolute and 9 relative alterations in immunological profile parameters of asymptomatic patients at the time of first pregnancy testing, that could present a very helpful tool in clinical practice to discover patients at risk even before first symptoms occur.

Another interesting point in screening for altered potential biomarkers is the fact that the discovered proteins in maternal plasma may help to identify potential therapeutical targets in the long run [5, 11]**.**

 Cytokines  play an important role in implantation and early pregnancy [16, 28]**.** For example, IL-1 receptors have been shown to be located on the early human embryo, emphasizing the important role of IL1-ra in early pregnancy [18]. Therefore, we aimed to establish chemokines as biomarkers at the time of pregnancy testing, some of which were found to be changed in patients with proven abortions.

Especially MIP1a, IL-1ra and TNF alpha showed promising results: not only absolute values were altered, additionally we could demonstrate altered relative expression changes, when put in relation to hCG, potentially making them even more predictive for pregnancy outcome in such an early phase.

MCP1 (aka CCL-2) is discussed controversially in the literature: it is considered to cause a shift towards Th2-response, which seems to present an important process in early pregnancy [23–25]. However, contrary to this observation, serum levels of MCP1 in early pregnancy were higher in women with RSA [26], whereas other studies did not see any significant alteration [5].

With our data we can rather support the observation that MCP1 does not show any change concerning the absolute values in patients with abortion during pregnancy. However, the *ratio* of MCP1/hCG varies between women with ongoing pregnancy and women suffering from abortion at the time of pregnancy testing already. This emphasizes the usefulness of the chemokine/hCG ratios compared to absolute levels.

TNF-alpha also favours a shift towards Th2-guided immune response. Despite this fact, our data does not answer the question which immunological pathway is dominant in early pregnancy or abortion.

Other factors, such as TPO or IL-6 that showed alterations at other time points in pregnancy [17, 20], were not significantly altered regarding the absolute expression at this very early time point in pregnancy, but showed altered expression when put in relation to hCG.

This observation may be contributed to the fact that absolute alterations may be already present at time of pregnancy testing but do not show an effect that allows to reach statistical significance with a small sample size.

To screen for promising biomarkers at this very early stage in pregnancy, we tent to enhance the prognostic marker for later abortion by examining a cytokines/ hCG correlation, leading to changes in clinical management. These ratios showed very promising results and were, to our knowledge, not described elsewhere in the literature. Due to the fact that two potentially altered proteins are included in this ratio, we think that these ratios are more predictive for pregnancy outcome than absolute cytokine or hCG expression levels and present therefore a high potential for the use as a biomarker. Furthermore, even though hCG levels were found to be significantly decreased in patients with abortion compared to those with ongoing pregnancy at the time of pregnancy testing, their levels are within normal limits and can therefore not be used as predictive factor of pregnancy outcome.

The limitations of this study are its small sample size and the fact, that we only detected protein in maternal plasma with no further validation technique. Therefore, our results require further validation after power calculation in order evaluate the different ratios. Furthermore, it is worth to investigate if an index involving different chemokines can be created.

Our study included patients after ART solely. We can’t exclude that these findings can be transmitted to patients who conceived spontaneously. However, as ART seems to influence cytokine profile, our findings can be specific for patients following in-vitro-fertilization [29].

## Conclusion

In conclusion, we have shown that cytokines in relation to hCG, as well as the ratio of GCSF/IL-1ra and MIP1a/TGF-beta1 after 4 weeks of gestation are significantly altered in women with abortion, indicating their potential use as a prognostic biomarker. Particularly the ratios of MIP1a, IL-1ra and TNF alpha to hCG show very promising results.

## Abbrevations

CCL: Chemokine (C-C Motif) ligand
ELISA: Enzyme-linked Immunosorbent Assay
GCSF: Granulocyte-colony stimulating factor
hCG: Human choriogonadotropin
ICSI: Intracytoplasmic sperm injection
IL: Interleukin
IVF: In vitro fertilization
MIP: Macrophage inflammatory protein
PAPP-A: Pregnancy associated plasma protein A
RSA: Recurrent spontaneous abortion
SEM: Standard error of mean
TGF: Transforming growth factor
TNF: Tumor necrosis factor
TPO: Thyreoperoxidase
